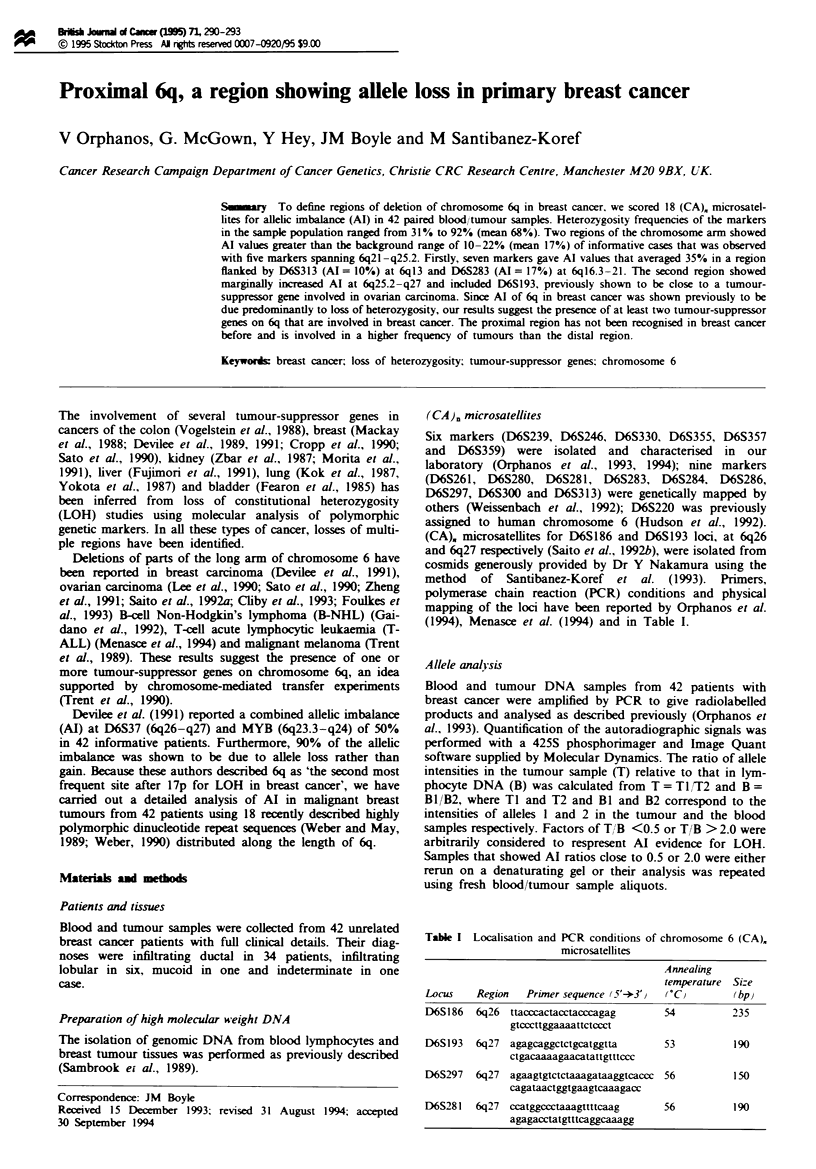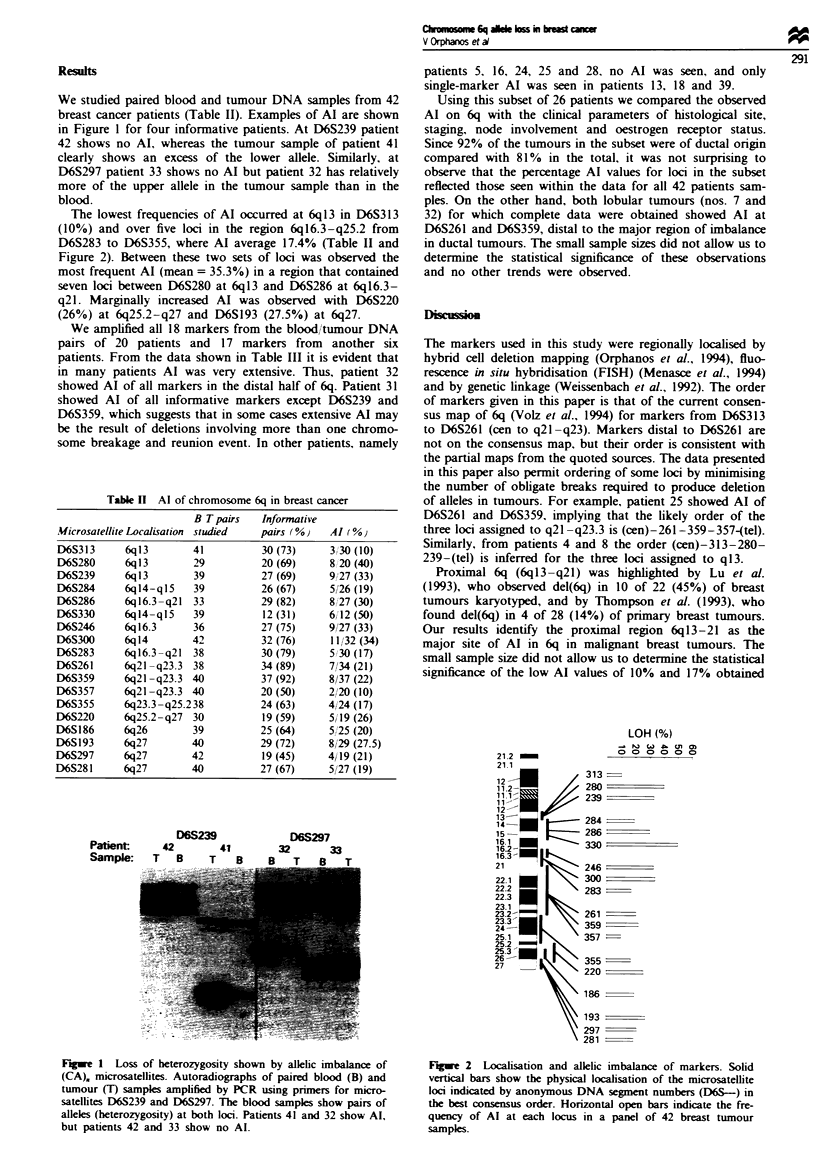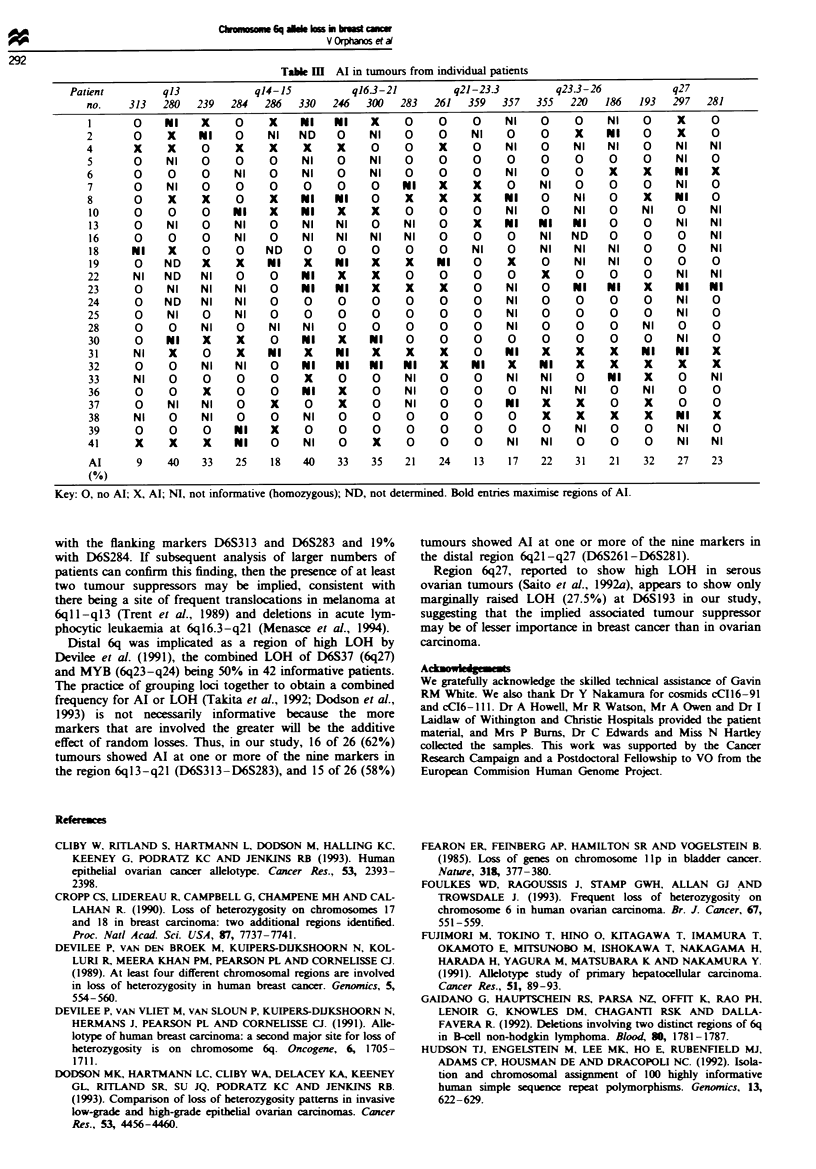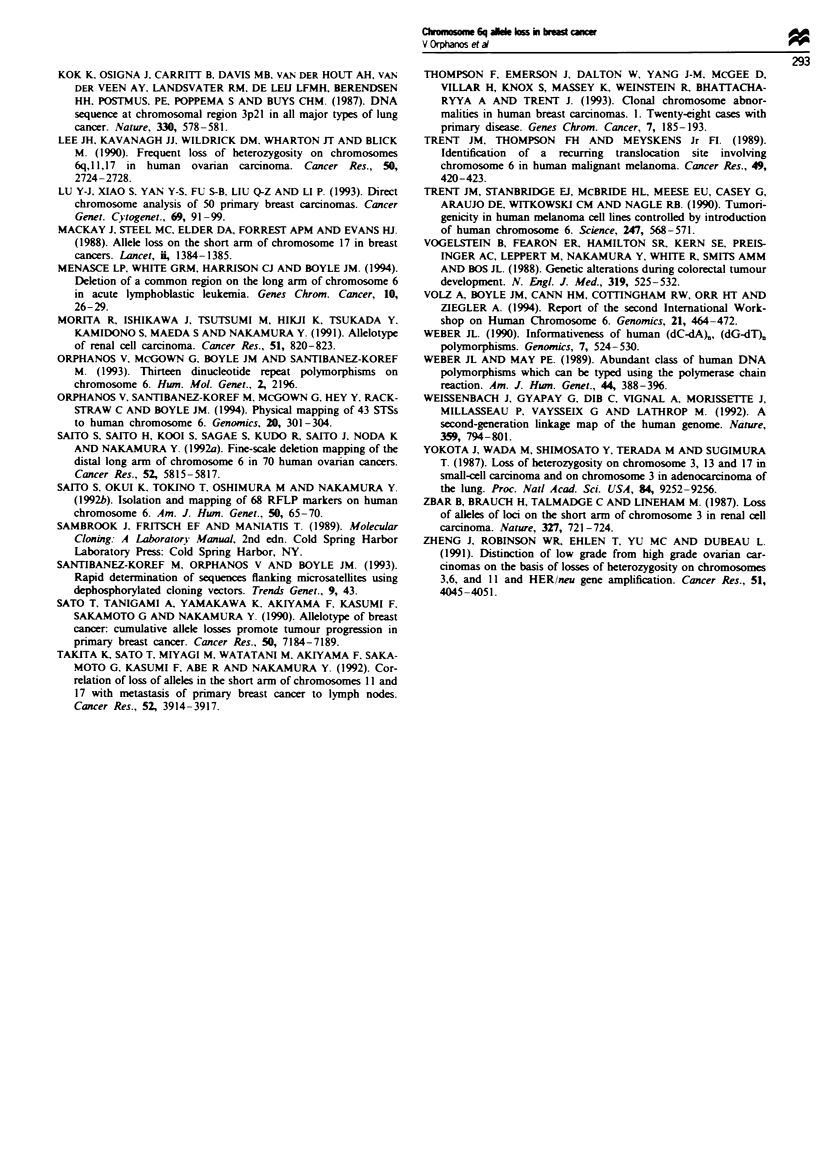# Proximal 6q, a region showing allele loss in primary breast cancer.

**DOI:** 10.1038/bjc.1995.58

**Published:** 1995-02

**Authors:** V. Orphanos, G. McGown, Y. Hey, J. M. Boyle, M. Santibanez-Koref

**Affiliations:** Cancer Research Campaign Department of Cancer Genetics, Christie CRC Research Centre, Manchester, UK.

## Abstract

**Images:**


British Journal of Cancer (1995) 71, 290-293

© 1995 Stockton Press All rights reserved 0007-0920/95 $9.00

### Proximal 6q, a region showing allele loss in primary breast cancer

V Orphanos, G. McGown, Y Hey, JM Boyle and M Santibanez-Koref

*Cancer Research Campaign Department of Cancer Genetics, Christie CRC Research Centre, Manchester M20 9BX, UK.*

**Summary** To define regions of deletion of chromosome 6q in breast cancer, we scored 18 $(CA)_n$ microsatellites for allelic imbalance (AI) in 42 paired blood tumour samples. Heterozygosity frequencies of the markers in the sample population ranged from 31% to 92% (mean 68%). Two regions of the chromosome arm showed AI values greater than the background range of 10-22% (mean 17%) of informative cases that was observed with five markers spanning 6q21-q25.2. Firstly, seven markers gave AI values that averaged 35% in a region flanked by D6S313 (AI = 10%) at 6q13 and D6S283 (AI = 17%) at 6q16.3-21. The second region showed marginally increased AI at 6q25.2-q27 and included D6S193, previously shown to be close to a tumour-suppressor gene involved in ovarian carcinoma. Since AI of 6q in breast cancer was shown previously to be due predominantly to loss of heterozygosity, our results suggest the presence of at least two tumour-suppressor genes on 6q that are involved in breast cancer. The proximal region has not been recognised in breast cancer before and is involved in a higher frequency of tumours than the distal region.

**Keywords:** breast cancer; loss of heterozygosity; tumour-suppressor genes; chromosome 6

The involvement of several tumour-suppressor genes in cancers of the colon (Vogelstein *et al.*, 1988), breast (Mackay *et al.*, 1988; Devilee *et al.*, 1989, 1991; Cropp *et al.*, 1990; Sato *et al.*, 1990), kidney (Zbar *et al.*, 1987; Morita *et al.*, 1991), liver (Fujimori *et al.*, 1991), lung (Kok *et al.*, 1987, Yokota *et al.*, 1987) and bladder (Fearon *et al.*, 1985) has been inferred from loss of constitutional heterozygosity (LOH) studies using molecular analysis of polymorphic genetic markers. In all these types of cancer, losses of multiple regions have been identified.

Deletions of parts of the long arm of chromosome 6 have been reported in breast carcinoma (Devilee *et al.*, 1991), ovarian carcinoma (Lee *et al.*, 1990; Sato *et al.*, 1990; Zheng *et al.*, 1991; Saito *et al.*, 1992*a*; Cliby *et al.*, 1993; Foulkes *et al.*, 1993) B-cell Non-Hodgkin's lymphoma (B-NHL) (Gaidano *et al.*, 1992), T-cell acute lymphocytic leukaemia (T-ALL) (Menasce *et al.*, 1994) and malignant melanoma (Trent *et al.*, 1989). These results suggest the presence of one or more tumour-suppressor genes on chromosome 6q, an idea supported by chromosome-mediated transfer experiments (Trent *et al.*, 1990).

Devilee *et al.* (1991) reported a combined allelic imbalance (AI) at D6S37 (6q26-q27) and MYB (6q23.3-q24) of 50% in 42 informative patients. Furthermore, 90% of the allelic imbalance was shown to be due to allele loss rather than gain. Because these authors described 6q as 'the second most frequent site after 17p for LOH in breast cancer', we have carried out a detailed analysis of AI in malignant breast tumours from 42 patients using 18 recently described highly polymorphic dinucleotide repeat sequences (Weber and May, 1989; Weber, 1990) distributed along the length of 6q.

### Materials and methods

### Patients and tissues

Blood and tumour samples were collected from 42 unrelated breast cancer patients with full clinical details. Their diagnoses were infiltrating ductal in 34 patients, infiltrating lobular in six, mucoid in one and indeterminate in one case.

### Preparation of high molecular weight DNA

The isolation of genomic DNA from blood lymphocytes and breast tumour tissues was performed as previously described (Sambrook *et al.*, 1989).

Correspondence: JM Boyle
Received 15 December 1993; revised 31 August 1994; accepted 30 September 1994

### $(CA)_n$ microsatellites

Six markers (D6S239, D6S246, D6S330, D6S355, D6S357 and D6S359) were isolated and characterised in our laboratory (Orphanos *et al.*, 1993, 1994); nine markers (D6S261, D6S280, D6S281, D6S283, D6S284, D6S286, D6S297, D6S300 and D6S313) were genetically mapped by others (Weissenbach *et al.*, 1992); D6S220 was previously assigned to human chromosome 6 (Hudson *et al.*, 1992). $(CA)_n$ microsatellites for D6S186 and D6S193 loci, at 6q26 and 6q27 respectively (Saito *et al.*, 1992*b*), were isolated from cosmids generously provided by Dr Y Nakamura using the method of Santibanez-Koref *et al.* (1993). Primers, polymerase chain reaction (PCR) conditions and physical mapping of the loci have been reported by Orphanos *et al.* (1994), Menasce *et al.* (1994) and in Table I.

### Allele analysis

Blood and tumour DNA samples from 42 patients with breast cancer were amplified by PCR to give radiolabelled products and analysed as described previously (Orphanos *et al.*, 1993). Quantification of the autoradiographic signals was performed with a 425S phosphorimager and Image Quant software supplied by Molecular Dynamics. The ratio of allele intensities in the tumour sample (T) relative to that in lymphocyte DNA (B) was calculated from T = T1 T2 and B = B1 B2, where T1 and T2 and B1 and B2 correspond to the intensities of alleles 1 and 2 in the tumour and the blood samples respectively. Factors of T B <0.5 or T B >2.0 were arbitrarily considered to respresent AI evidence for LOH. Samples that showed AI ratios close to 0.5 or 2.0 were either rerun on a denaturating gel or their analysis was repeated using fresh blood tumour sample aliquots.

**Table I** Localisation and PCR conditions of chromosome 6 $(CA)_n$ microsatellites

| *Locus* | *Region* | *Primer sequence (5'→3')* | *Annealing temperature (°C)* | *Size (bp)* |
|---|---|---|---|---|
| D6S186 | 6q26 | ttacccactacctacccagag<br>gtcccttggaaaattctccct | 54 | 235 |
| D6S193 | 6q27 | agagcaggctctgcatggtta<br>ctgacaaaagaacatattgtttccc | 53 | 190 |
| D6S297 | 6q27 | agaagtgtctctaaagataaggtcaccc<br>cagataactggtgaagtcaaagacc | 56 | 150 |
| D6S281 | 6q27 | ccatggccctaaagttttcaag<br>agagacctatgtttcaggcaaagg | 56 | 190 |

### Results

We studied paired blood and tumour DNA samples from 42 breast cancer patients (Table II). Examples of AI are shown in Figure 1 for four informative patients. At D6S239 patient 42 shows no AI, whereas the tumour sample of patient 41 clearly shows an excess of the lower allele. Similarly, at D6S297 patient 33 shows no AI but patient 32 has relatively more of the upper allele in the tumour sample than in the blood.

The lowest frequencies of AI occurred at 6q13 in D6S313 (10%) and over five loci in the region 6q16.3–q25.2 from D6S283 to D6S355, where AI average 17.4% (Table II and Figure 2). Between these two sets of loci was observed the most frequent AI (mean = 35.3%) in a region that contained seven loci between D6S280 at 6q13 and D6S286 at 6q16.3–q21. Marginally increased AI was observed with D6S220 (26%) at 6q25.2–q27 and D6S193 (27.5%) at 6q27.

We amplified all 18 markers from the blood/tumour DNA pairs of 20 patients and 17 markers from another six patients. From the data shown in Table III it is evident that in many patients AI was very extensive. Thus, patient 32 showed AI of all markers in the distal half of 6q. Patient 31 showed AI of all informative markers except D6S239 and D6S359, which suggests that in some cases extensive AI may be the result of deletions involving more than one chromosome breakage and reunion event. In other patients, namely patients 5, 16, 24, 25 and 28, no AI was seen, and only single-marker AI was seen in patients 13, 18 and 39.

Using this subset of 26 patients we compared the observed AI on 6q with the clinical parameters of histological site, staging, node involvement and oestrogen receptor status. Since 92% of the tumours in the subset were of ductal origin compared with 81% in the total, it was not surprising to observe that the percentage AI values for loci in the subset reflected those seen within the data for all 42 patients samples. On the other hand, both lobular tumours (nos. 7 and 32) for which complete data were obtained showed AI at D6S261 and D6S359, distal to the major region of imbalance in ductal tumours. The small sample sizes did not allow us to determine the statistical significance of these observations and no other trends were observed.

**Table II** AI of chromosome 6q in breast cancer

| *Microsatellite* | *Localisation* | *B T pairs studied* | *Informative pairs (%)* | *AI (%)* |
|---|---|---|---|---|
| D6S313 | 6q13 | 41 | 30 (73) | 3/30 (10) |
| D6S280 | 6q13 | 29 | 20 (69) | 8/20 (40) |
| D6S239 | 6q13 | 39 | 27 (69) | 9/27 (33) |
| D6S284 | 6q14–q15 | 39 | 26 (67) | 5/26 (19) |
| D6S286 | 6q16.3–q21 | 33 | 29 (82) | 8/27 (30) |
| D6S330 | 6q14–q15 | 39 | 12 (31) | 6/12 (50) |
| D6S246 | 6q16.3 | 36 | 27 (75) | 9/27 (33) |
| D6S300 | 6q14 | 42 | 32 (76) | 11/32 (34) |
| D6S283 | 6q16.3–q21 | 38 | 30 (79) | 5/30 (17) |
| D6S261 | 6q21–q23.3 | 38 | 34 (89) | 7/34 (21) |
| D6S359 | 6q21–q23.3 | 40 | 37 (92) | 8/37 (22) |
| D6S357 | 6q21–q23.3 | 40 | 20 (50) | 2/20 (10) |
| D6S355 | 6q23.3–q25.2 | 38 | 24 (63) | 4/24 (17) |
| D6S220 | 6q25.2–q27 | 30 | 19 (59) | 5/19 (26) |
| D6S186 | 6q26 | 39 | 25 (64) | 5/25 (20) |
| D6S193 | 6q27 | 40 | 29 (72) | 8/29 (27.5) |
| D6S297 | 6q27 | 42 | 19 (45) | 4/19 (21) |
| D6S281 | 6q27 | 40 | 27 (67) | 5/27 (19) |

**Figure 1** Loss of heterozygosity shown by allelic imbalance of $(CA)_n$ microsatellites. Autoradiographs of paired blood (B) and tumour (T) samples amplified by PCR using primers for microsatellites D6S239 and D6S297. The blood samples show pairs of alleles (heterozygosity) at both loci. Patients 41 and 32 show AI, but patients 42 and 33 show no AI.

### Discussion

The markers used in this study were regionally localised by hybrid cell deletion mapping (Orphanos *et al.*, 1994), fluorescence *in situ* hybridisation (FISH) (Menasce *et al.*, 1994) and by genetic linkage (Weissenbach *et al.*, 1992). The order of markers given in this paper is that of the current consensus map of 6q (Volz *et al.*, 1994) for markers from D6S313 to D6S261 (cen to q21–q23). Markers distal to D6S261 are not on the consensus map, but their order is consistent with the partial maps from the quoted sources. The data presented in this paper also permit ordering of some loci by minimising the number of obligate breaks required to produce deletion of alleles in tumours. For example, patient 25 showed AI of D6S261 and D6S359, implying that the likely order of the three loci assigned to q21–q23.3 is (cen)–261–359–357–(tel). Similarly, from patients 4 and 8 the order (cen)–313–280–239–(tel) is inferred for the three loci assigned to q13.

Proximal 6q (6q13–q21) was highlighted by Lu *et al.* (1993), who observed del(6q) in 10 of 22 (45%) of breast tumours karyotyped, and by Thompson *et al.* (1993), who found del(6q) in 4 of 28 (14%) of primary breast tumours. Our results identify the proximal region 6q13–21 as the major site of AI in 6q in malignant breast tumours. The small sample size did not allow us to determine the statistical significance of the low AI values of 10% and 17% obtained

**Figure 2** Localisation and allelic imbalance of markers. Solid vertical bars show the physical localisation of the microsatellite loci indicated by anonymous DNA segment numbers (D6S—) in the best consensus order. Horizontal open bars indicate the frequency of AI at each locus in a panel of 42 breast tumour samples.

**Table III** AI in tumours from individual patients

| *Patient* | | *q13* | | | *q14–15* | | | *q16.3–21* | | | *q21–23.3* | | | *q23.3–26* | | | *q27* | |
|---|---|---|---|---|---|---|---|---|---|---|---|---|---|---|---|---|---|---|
| *no.* | *313* | *280* | *239* | *284* | *286* | *330* | *246* | *300* | *283* | *261* | *359* | *357* | *355* | *220* | *186* | *193* | *297* | *281* |
| 1 | O | **NI** | X | O | X | **NI** | **NI** | X | O | O | O | NI | O | O | NI | O | X | O |
| 2 | O | X | **NI** | O | NI | ND | O | NI | O | O | NI | O | O | X | **NI** | O | X | O |
| 4 | X | X | O | X | X | X | X | O | O | X | O | NI | O | NI | NI | O | NI | NI |
| 5 | O | NI | O | O | O | NI | O | NI | O | O | O | O | O | O | O | O | NI | O |
| 6 | O | O | O | NI | O | NI | O | NI | O | O | O | NI | O | O | X | X | **NI** | X |
| 7 | O | NI | O | O | O | O | O | O | **NI** | X | X | O | NI | O | O | O | NI | O |
| 8 | O | X | X | O | X | **NI** | **NI** | O | X | X | X | **NI** | O | NI | O | X | **NI** | O |
| 10 | O | O | O | **NI** | X | **NI** | X | X | O | O | O | NI | O | NI | O | NI | O | NI |
| 13 | O | NI | O | NI | O | NI | NI | O | NI | O | X | **NI** | **NI** | **NI** | O | O | NI | NI |
| 16 | O | O | O | NI | O | NI | NI | NI | NI | O | O | O | NI | ND | O | O | O | NI |
| 18 | **NI** | X | O | O | ND | O | O | O | O | O | NI | O | NI | NI | NI | O | O | NI |
| 19 | O | ND | X | X | **NI** | X | **NI** | X | X | **NI** | O | X | O | NI | NI | O | O | O |
| 22 | NI | ND | NI | O | O | **NI** | X | X | O | O | O | O | X | O | O | O | NI | NI |
| 23 | O | NI | NI | NI | O | **NI** | **NI** | X | X | X | O | NI | O | **NI** | **NI** | X | **NI** | **NI** |
| 24 | O | ND | NI | NI | O | O | O | O | O | O | O | NI | O | O | O | O | NI | O |
| 25 | O | NI | O | NI | O | O | O | O | O | O | O | NI | O | O | O | O | NI | O |
| 28 | O | O | NI | O | NI | NI | O | O | O | O | O | NI | O | O | O | NI | O | O |
| 30 | O | **NI** | X | X | O | **NI** | X | **NI** | O | O | O | O | O | O | O | O | NI | O |
| 31 | NI | X | O | X | **NI** | X | **NI** | X | X | X | O | **NI** | X | X | X | **NI** | **NI** | X |
| 32 | O | O | NI | NI | O | **NI** | **NI** | **NI** | **NI** | X | **NI** | X | **NI** | X | X | X | X | X |
| 33 | NI | O | O | O | O | X | O | O | NI | O | O | NI | NI | O | **NI** | X | O | NI |
| 36 | O | O | X | O | O | **NI** | X | O | NI | O | O | O | NI | NI | O | NI | O | O |
| 37 | O | NI | NI | O | X | O | X | O | NI | O | O | **NI** | X | X | O | X | O | O |
| 38 | NI | O | NI | O | O | NI | O | O | O | O | O | O | X | X | X | X | **NI** | X |
| 39 | O | O | O | **NI** | X | O | O | O | O | O | O | O | O | NI | O | O | NI | O |
| 41 | X | X | X | **NI** | O | NI | O | X | O | O | O | NI | NI | O | O | O | NI | NI |
| AI (%) | 9 | 40 | 33 | 25 | 18 | 40 | 33 | 35 | 21 | 24 | 13 | 17 | 22 | 31 | 21 | 32 | 27 | 23 |

Key: O, no AI; X, AI; NI, not informative (homozygous); ND, not determined. Bold entries maximise regions of AI.

with the flanking markers D6S313 and D6S283 and 19% with D6S284. If subsequent analysis of larger numbers of patients can confirm this finding, then the presence of at least two tumour suppressors may be implied, consistent with there being a site of frequent translocations in melanoma at 6q11–q13 (Trent *et al.*, 1989) and deletions in acute lymphocytic leukaemia at 6q16.3–q21 (Menasce *et al.*, 1994).

Distal 6q was implicated as a region of high LOH by Devilee *et al.* (1991), the combined LOH of D6S37 (6q27) and MYB (6q23–q24) being 50% in 42 informative patients. The practice of grouping loci together to obtain a combined frequency for AI or LOH (Takita *et al.*, 1992; Dodson *et al.*, 1993) is not necessarily informative because the more markers that are involved the greater will be the additive effect of random losses. Thus, in our study, 16 of 26 (62%) tumours showed AI at one or more of the nine markers in the region 6q13–q21 (D6S313–D6S283), and 15 of 26 (58%) tumours showed AI at one or more of the nine markers in the distal region 6q21–q27 (D6S261–D6S281).

Region 6q27, reported to show high LOH in serous ovarian tumours (Saito *et al.*, 1992*a*), appears to show only marginally raised LOH (27.5%) at D6S193 in our study, suggesting that the implied associated tumour suppressor may be of lesser importance in breast cancer than in ovarian carcinoma.

**Acknowledgements**

We gratefully acknowledge the skilled technical assistance of Gavin RM White. We also thank Dr Y Nakamura for cosmids cCI16-91 and cCI6-111. Dr A Howell, Mr R Watson, Mr A Owen and Dr I Laidlaw of Withington and Christie Hospitals provided the patient material, and Mrs P Burns, Dr C Edwards and Miss N Hartley collected the samples. This work was supported by the Cancer Research Campaign and a Postdoctoral Fellowship to VO from the European Commision Human Genome Project.

### References

CLIBY W, RITLAND S, HARTMANN L, DODSON M, HALLING KC, KEENEY G, PODRATZ KC AND JENKINS RB (1993). Human epithelial ovarian cancer allelotype. *Cancer Res.*, **53**, 2393–2398.

CROPP CS, LIDEREAU R, CAMPBELL G, CHAMPENE MH AND CALLAHAN R. (1990). Loss of heterozygosity on chromosomes 17 and 18 in breast carcinoma: two additional regions identified. *Proc. Natl Acad. Sci. USA*, **87**, 7737–7741.

DEVILEE P, VAN DEN BROEK M, KUIPERS-DIJKSHOORN N, KOLLURI R, MEERA KHAN PM, PEARSON PL AND CORNELISSE CJ. (1989). At least four different chromosomal regions are involved in loss of heterozygosity in human breast cancer. *Genomics*, **5**, 554–560.

DEVILEE P, VAN VLIET M, VAN SLOUN P, KUIPERS-DIJKSHOORN N, HERMANS J, PEARSON PL AND CORNELISSE CJ. (1991). Allelotype of human breast carcinoma: a second major site for loss of heterozygosity is on chromosome 6q. *Oncogene*, **6**, 1705–1711.

DODSON MK, HARTMANN LC, CLIBY WA, DELACEY KA, KEENEY GL, RITLAND SR, SU JQ, PODRATZ KC AND JENKINS RB. (1993). Comparison of loss of heterozygosity patterns in invasive low-grade and high-grade epithelial ovarian carcinomas. *Cancer Res.*, **53**, 4456–4460.

FEARON ER, FEINBERG AP, HAMILTON SR AND VOGELSTEIN B. (1985). Loss of genes on chromosome 11p in bladder cancer. *Nature*, **318**, 377–380.

FOULKES WD, RAGOUSSIS J, STAMP GWH, ALLAN GJ AND TROWSDALE J. (1993). Frequent loss of heterozygosity on chromosome 6 in human ovarian carcinoma. *Br. J. Cancer*, **67**, 551–559.

FUJIMORI M, TOKINO T, HINO O, KITAGAWA T, IMAMURA T, OKAMOTO E, MITSUNOBO M, ISHOKAWA T, NAKAGAMA H, HARADA H, YAGURA M, MATSUBARA K AND NAKAMURA Y. (1991). Allelotype study of primary hepatocellular carcinoma. *Cancer Res.*, **51**, 89–93.

GAIDANO G, HAUPTSCHEIN RS, PARSA NZ, OFFIT K, RAO PH, LENOIR G, KNOWLES DM, CHAGANTI RSK AND DALLA-FAVERA R. (1992). Deletions involving two distinct regions of 6q in B-cell non-hodgkin lymphoma. *Blood*, **80**, 1781–1787.

HUDSON TJ, ENGELSTEIN M, LEE MK, HO E, RUBENFIELD MJ, ADAMS CP, HOUSMAN DE AND DRACOPOLI NC. (1992). Isolation and chromosomal assignment of 100 highly informative human simple sequence repeat polymorphisms. *Genomics*, **13**, 622–629.

KOK K, OSIGNA J, CARRITT B, DAVIS MB, VAN DER HOUT AH, VAN DER VEEN AY, LANDSVATER RM, DE LEIJ LFMH, BERENDSEN HH, POSTMUS, PE, POPPEMA S AND BUYS CHM. (1987). DNA sequence at chromosomal region 3p21 in all major types of lung cancer. *Nature*, **330**, 578-581.

LEE JH, KAVANAGH JJ, WILDRICK DM, WHARTON JT AND BLICK M. (1990). Frequent loss of heterozygosity on chromosomes 6q,11,17 in human ovarian carcinoma. *Cancer Res.*, **50**, 2724-2728.

LU Y-J, XIAO S, YAN Y-S, FU S-B, LIU Q-Z AND LI P. (1993). Direct chromosome analysis of 50 primary breast carcinomas. *Cancer Genet. Cytogenet.*, **69**, 91-99.

MACKAY J, STEEL MC, ELDER DA, FORREST APM AND EVANS HJ. (1988). Allele loss on the short arm of chromosome 17 in breast cancers. *Lancet*, **ii**, 1384-1385.

MENASCE LP, WHITE GRM, HARRISON CJ AND BOYLE JM. (1994). Deletion of a common region on the long arm of chromosome 6 in acute lymphoblastic leukemia. *Genes Chrom. Cancer*, **10**, 26-29.

MORITA R, ISHIKAWA J, TSUTSUMI M, HIKJI K, TSUKADA Y, KAMIDONO S, MAEDA S AND NAKAMURA Y. (1991). Allelotype of renal cell carcinoma. *Cancer Res.*, **51**, 820-823.

ORPHANOS V, McGOWN G, BOYLE JM AND SANTIBANEZ-KOREF M. (1993). Thirteen dinucleotide repeat polymorphisms on chromosome 6. *Hum. Mol. Genet.*, **2**, 2196.

ORPHANOS V, SANTIBANEZ-KOREF M, McGOWN G, HEY Y, RACK-STRAW C AND BOYLE JM. (1994). Physical mapping of 43 STSs to human chromosome 6. *Genomics*, **20**, 301-304.

SAITO S, SAITO H, KOOI S, SAGAE S, KUDO R, SAITO J, NODA K AND NAKAMURA Y. (1992*a*). Fine-scale deletion mapping of the distal long arm of chromosome 6 in 70 human ovarian cancers. *Cancer Res.*, **52**, 5815-5817.

SAITO S, OKUI K, TOKINO T, OSHIMURA M AND NAKAMURA Y. (1992*b*). Isolation and mapping of 68 RFLP markers on human chromosome 6. *Am. J. Hum. Genet.*, **50**, 65-70.

SAMBROOK J, FRITSCH EF AND MANIATIS T. (1989). *Molecular Cloning: A Laboratory Manual*, 2nd edn. Cold Spring Harbor Laboratory Press: Cold Spring Harbor, NY.

SANTIBANEZ-KOREF M, ORPHANOS V AND BOYLE JM. (1993). Rapid determination of sequences flanking microsatellites using dephosphorylated cloning vectors. *Trends Genet.*, **9**, 43.

SATO T, TANIGAMI A, YAMAKAWA K, AKIYAMA F, KASUMI F, SAKAMOTO G AND NAKAMURA Y. (1990). Allelotype of breast cancer: cumulative allele losses promote tumour progression in primary breast cancer. *Cancer Res.*, **50**, 7184-7189.

TAKITA K, SATO T, MIYAGI M, WATATANI M, AKIYAMA F, SAKAMOTO G, KASUMI F, ABE R AND NAKAMURA Y. (1992). Correlation of loss of alleles in the short arm of chromosomes 11 and 17 with metastasis of primary breast cancer to lymph nodes. *Cancer Res.*, **52**, 3914-3917.

THOMPSON F, EMERSON J, DALTON W, YANG J-M, McGEE D, VILLAR H, KNOX S, MASSEY K, WEINSTEIN R, BHATTACHARYYA A AND TRENT J. (1993). Clonal chromosome abnormalities in human breast carcinomas. 1. Twenty-eight cases with primary disease. *Genes Chrom. Cancer*, **7**, 185-193.

TRENT JM, THOMPSON FH AND MEYSKENS Jr FI. (1989). Identification of a recurring translocation site involving chromosome 6 in human malignant melanoma. *Cancer Res.*, **49**, 420-423.

TRENT JM, STANBRIDGE EJ, McBRIDE HL, MEESE EU, CASEY G, ARAUJO DE, WITKOWSKI CM AND NAGLE RB. (1990). Tumorigenicity in human melanoma cell lines controlled by introduction of human chromosome 6. *Science*, **247**, 568-571.

VOGELSTEIN B, FEARON ER, HAMILTON SR, KERN SE, PREISINGER AC, LEPPERT M, NAKAMURA Y, WHITE R, SMITS AMM AND BOS JL. (1988). Genetic alterations during colorectal tumour development. *N. Engl. J. Med.*, **319**, 525-532.

VOLZ A, BOYLE JM, CANN HM, COTTINGHAM RW, ORR HT AND ZIEGLER A. (1994). Report of the second International Workshop on Human Chromosome 6. *Genomics*, **21**, 464-472.

WEBER JL. (1990). Informativeness of human $(dC\text{-}dA)_n$, $(dG\text{-}dT)_n$ polymorphisms. *Genomics*, **7**, 524-530.

WEBER JL AND MAY PE. (1989). Abundant class of human DNA polymorphisms which can be typed using the polymerase chain reaction. *Am. J. Hum. Genet.*, **44**, 388-396.

WEISSENBACH J, GYAPAY G, DIB C, VIGNAL A, MORISSETTE J, MILLASSEAU P, VAYSSEIX G AND LATHROP M. (1992). A second-generation linkage map of the human genome. *Nature*, **359**, 794-801.

YOKOTA J, WADA M, SHIMOSATO Y, TERADA M AND SUGIMURA T. (1987). Loss of heterozygosity on chromosome 3, 13 and 17 in small-cell carcinoma and on chromosome 3 in adenocarcinoma of the lung. *Proc. Natl Acad. Sci. USA*, **84**, 9252-9256.

ZBAR B, BRAUCH H, TALMADGE C AND LINEHAM M. (1987). Loss of alleles of loci on the short arm of chromosome 3 in renal cell carcinoma. *Nature*, **327**, 721-724.

ZHENG J, ROBINSON WR, EHLEN T, YU MC AND DUBEAU L. (1991). Distinction of low grade from high grade ovarian carcinomas on the basis of losses of heterozygosity on chromosomes 3,6, and 11 and HER/*neu* gene amplification. *Cancer Res.*, **51**, 4045-4051.